# Challenges in estimating virus divergence times in short epidemic timescales with special reference to the evolution of SARS-CoV-2 pandemic

**DOI:** 10.1590/1678-4685-GMB-2020-0254

**Published:** 2021-02-08

**Authors:** Carlos G. Schrago, Lucia P. Barzilai

**Affiliations:** 1 Universidade Federal do Rio de Janeiro, Departamento de Genética, Rio de Janeiro, RJ, Brazil

**Keywords:** Coronavirus, evolution, transmission, simulation, tree shape

## Abstract

The estimation of evolutionary parameters provides essential information for designing public health policies. In short time intervals, however, nucleotide substitutions are ineffective to record all complexities of virus population dynamics. In this sense, the current SARS-CoV-2 pandemic poses a challenge for evolutionary analysis. We used computer simulation to evolve populations in scenarios of varying temporal intervals to evaluate the impact of the age of an epidemic on estimates of time and geography. Before estimating virus timescales, the shape of tree topologies can be used as a proxy to assess the effectiveness of the virus phylogeny in providing accurate estimates of evolutionary parameters. In short timescales, estimates have larger uncertainty. We compared the predictions from simulations with empirical data. The tree shape of SARS-CoV-2 was closer to shorter timescales scenarios, which yielded parametric estimates with larger uncertainty, suggesting that estimates from these datasets should be evaluated cautiously. To increase the accuracy of the estimates of virus transmission times between populations, the uncertainties associated with the age estimates of both the crown and stem nodes should be communicated. We place the age of the common ancestor of the current SARS-CoV-2 pandemic in late September 2019, corroborating an earlier emergence of the virus.

## Introduction

The evolutionary analysis of virus genomes frequently relies on molecular phylogenies, which illustrate the ancestry of lineages in tree graphs (Holmes, 2008). When trees are rooted, a time direction, implying ancestor-to-descendent relationship, is incorporated into phylogenies. Although rooted topologies are time-oriented, branch lengths are not necessarily proportional to absolute time units (Felsenstein, 2004). In order to fully incorporate the temporal dimension onto trees, divergence times of nodes must be estimated. When genetic divergences between genomes are linearly related to the age in which genomes shared a common ancestor, this task is straightforward (Kumar, 2005). By employing some calibration information, a direct linear transformation may be readily applied. This is the standard molecular clock, in which sequence substitution rates are constant along branches and across lineages. However, since the 1970s, rate constancy was found to be the exception rather than the rule (Langley and Fitch, 1974; Gillespie and Langley, 1979). Alternatively, timescales may be inferred by accommodating rate variation among lineages (Gillespie, 1991; Kishino *et al*., 2001; Bromham *et al*., 2018). 

Different approaches were proposed to handle rate heterogeneity in order to carry out molecular dating of sequence divergences. They can be roughly categorized into smoothing methods and methods that employ explicit models of substitution rate evolution in a Bayesian framework (Thorne *et al*., 1998; Sanderson, 2002; Drummond *et al*., 2005; Drummond *et al*., 2006; Smith and O’Meara, 2012). Bayesian methods require that probability density distributions are used as priors for calculating posterior distribution of parameters. For intraspecific population-level virus diversity, node ages of phylogenies are probabilistically described by the expected waiting times of the coalescent process (Kingman, 1982; Biek *et al*., 2015). Given the extent of the premises adopted, it is not surprising that dating of virus timescales is impacted by numerous factors (Stadler, 2009; Stadler and Yang, 2013).

**Figure 1 -:**
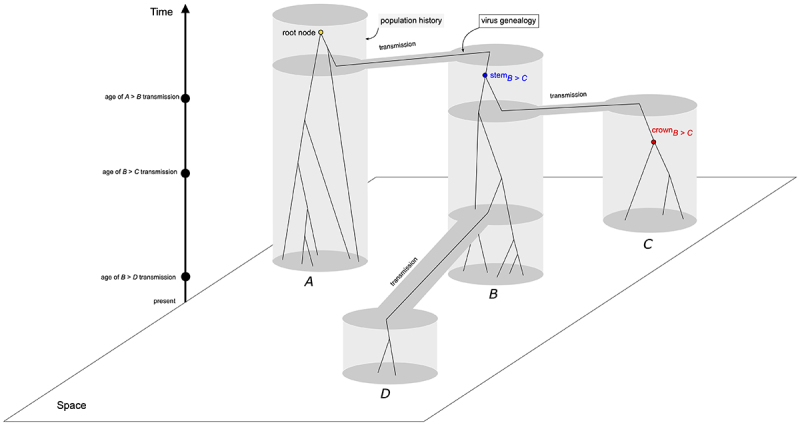
Evolutionary history of four populations (gray areas) and virus genealogies under the coalescent process (solid lines of descent). Grey areas delimit each population (*A*-*D*), exhibiting their historical relationship and transmission times - population in area *B* was founded from a single genome sampled from area *A*; populations from areas *C* and *D* were founded from genomes sampled from area *B* at different times. Although spatial relationship between populations were the same for all simulated scenarios (*10Y*, *2Y*, *6M*, and *1M*), sampling times, as well as the age of the root node, i.e., the common ancestor, varied accordingly. Within the population tree, a virus genealogy, modeled by the coalescent process depicts the transmission of genomes between areas (gene flow), and highlights the difference between the coalescent events of within-population genetic diversity (crown node), and between-populations genetic diversity (stem node).

One of such factors is the age that the virus population is circulating in the host species after the initial infection. Although frequently ignored, the age of the circulating virus population will affect the sampling strategy required for accurate inference of evolutionary parameters. Depending on the mutation rate, virus populations that successfully infected a new host species may not accumulate enough substitutions to allow for phylogenetic inference within a few generations after the initial transmission, resulting in branch lengths close to zero, and an large number of duplicated sequences (Boskova and Stadler, 2020). In a longer timescale, nucleotide substitutions shared by allelic lineages arise and the tree-like hierarchical relationship between sequences also emerges. The absence of the tree-like structure between sequences is a consequence of both the mutation rate and the time duration of internal branches and not of the coalescent process that model virus genealogies, i.e., the lines of descent (Figure 1). For instance, an interval between coalescent events of 10 generations is equivalent to a branch length close to zero substitutions/site in reconstructed phylogenies, even assuming the elevated mutation rates of RNA viruses.

Another factor impacting the estimation of virus timescales is the mismatch between the virus transmission history between populations and the genealogy of virus genomes. When transmission events along geographic areas take place, the sampling of the genetic diversity of the donor virus population is often incomplete and, because of the stochasticity of the coalescent process, both the ages of common ancestors of genomes and the monophyly of the virus genetic diversity in geographic areas is affected (Figure 1). This probability of mismatch between both trees (population history and gene genealogy) is increased if the time interval between transmission events is short and the level of genetic diversity is high (Tajima, 1983; Pamilo and Nei, 1988). This problem is equivalent to the mismatch between the species phylogeny and the gene tree in molecular phylogenetics, which is modeled by the multispecies coalescent (Liu *et al*., 2009; Degnan and Rosenberg, 2009), and will impact the estimates of the age of the epidemic as well as the recovery of the true pattern of the virus spread in space.

The current SARS-CoV-2 pandemic is an example of a recent zoonotic transmission, and an increasing number of studies has addressed the evolutionary dynamics of the novel coronavirus (Boni *et al*., 2020; Zhou *et al*., 2020). Despite the efforts, the age of the common ancestor of SARS-CoV-2, as well as the age of the split between the novel coronavirus and its sister lineage, is uncertain. For instance, Boni *et al*. (2020) used alignments free of recombining regions and employed an evolutionary rate prior based on MERS-CoV and HCoV-OC43 substitution rates. The divergence time between SARS-CoV-2 and RaTG13, its closest sister lineage sequenced so far, was estimated at years 1969, 1982, and 1948, depending on the genomic region analyzed. However, confidence intervals between these inferences were large. Most studies so far places the time to the most recent common ancestor (TMRCA) of circulating SARS-CoV-2 in November or December 2019, although confidence intervals extends from late September to late December 2019 (Biggerstaff *et al*., 2020). Lai *et al.* (2020), compared the performance of strict versus relaxed molecular clocks to estimate the age of the common ancestor of 52 SARS-CoV-2 sequences, and obtained 18 Nov. of 2019, with 95% credibility interval ranging from 10 Sept. 2019 to 28 Dec. 2019, as the most likely date. Candido *et al*. (2020) also estimated the TMRCA of SARS-CoV-2 in mid-November. Li *et al*. (2020), by analyzing 313 genomes, dated the emergence of SARS-CoV-2 in 11 Dec. 2019 (21 Nov. 2019 - 24 Dec. 2019), which is closer to the estimate of Zhang *et al*. (2020) obtained from 24 genomes (05 Dec 2019 to 23 Dec 2019). Moreover, the estimates of the rate of evolution also varied between studies, from 7.8 x 10^−4^ substitutions/site/year (s/s/y) (Boni *et al*., 2020; Lai *et al*., 2020) to 1.69 x 10^−3^ (Boni *et al*., 2020) and 2.24 x 10^−3^ s/s/y (Li *et al*., 2020).

We investigated the extent to which the age of the of the virus epidemic affects the inference of evolutionary parameters, in order to elucidate whether the discrepancies between estimates of SARS-CoV-2 timescales may be caused by the stochasticity of coalescent process and the reduced genetic diversity in narrow timescales. For the sake of comparison and validation of our methodological approach, we also investigated empirical data from other viruses that circulate in human populations along different timescales. Our approach compared the predictions from simulations with empirical data sets. We simulated sequences under different epidemic timescales to provide parametric values to be compared with the results from empirical virus datasets that exemplify both long-term and short-term epidemic scales. Comparisons were carried out using tree shape, as measured by the spectral density of tree topologies, which were calculated directly from undated maximum likelihood trees.

## Material and Methods

### Simulation to evaluate the effects of ILS and range of sampling time

To demonstrate the impact of the range of sampling times on the estimates of evolutionary parameters, we evolved sequences under varying evolutionary timescales. We incorporated two dimensions in our simulation - time and space - by allowing transmissions of lineages into new areas (Figure 1). Three parameters were investigated: the age of the epidemic (the root node), the ages of the transmission events, and geographical association, which are the main parameters inferred by most studies of virus evolution, with consequences for designing health policies. Our simulations were implemented using R scripts and consisted of populations that evolved under the standard neutral model, using Wright-Fisher sampling of haploid individual genes of 1500 bp; this sequence length has been shown sufficient to control for the effects of nucleotide sampling errors (Yang and Rannala, 2006). At each generation, which was equal to one day in our simulation, sites were mutated at a rate of 3 x 10^-8^ substitutions/site under the Jukes-Cantor model. This rate is equivalent to a per year rate of 1 x 10^-5^ s/s, which is the average rate for RNA viruses. The effective population sizes were set to 1000 individuals. Although this value is arguably smaller than empirical virus population sizes, it is appropriate for the computational demands of forward-time simulations that aim to generate tree topology shapes from short-term evolutionary dynamics.

All simulations started with a single population at area *A*. After a predetermined number of generations, which varied according to the time range in each scenario, a transmission event took place and a single allele was transmitted to area *B*. From area *B*, transmissions events also occurred to areas *C* and *D*. All transmissions consisted of unique events and no recurrent contact between areas were allowed (Figure 1).

This simple simulation allowed the investigation of the evolutionary parameters in different epidemiological timescales. Four scenarios were investigated by varying the total temporal extent of the epidemy, which equaled the age of the population from area *A*. In the first scenario, henceforth referred to *10Y*, the age of the common ancestor (tmrca) of the population from area *A* was 10 years. The transmission event to area *B* took place 8 years ago, while transmissions from *B* to areas *C* and *D* occurred at 6 and one year ago, respectively. In the second scenario, *2Y*, the common ancestor of population *A* was 2 years old, and the transmission to area *B* occurred 5 months later. From population *B*, transmissions to areas *B* and *C* took place at 1 year and 6 months ago, respectively. In the remaining two scenarios, we simulated the rapid geographic spread of a virus within less than one year. In scenario *6M*, the age of population of *A* was 6 months, the transmission to *B* occurred three months later, and transmissions from *B* to *C* and *D* occurred at 3 and 1 month ago respectively. Finally, in scenario *1M*, all transmissions took place within a single month (age of *A*): from *A* to *B* at 20 days ago; from *B* to *C* and *D* at 10 and 5 days ago respectively. For each scenario, we evolved 300 independent replicates. In each replicate, sequences were sampled serially along time intervals to yield approximately 28 sequences. This number was chosen to speed up computational time while ensuring the robustness of the results.

Phylogenies of each simulated alignment was estimated in IQ-TREE 1.6 (Nguyen *et al*., 2014) under the maximum likelihood framework employing the substitution model chosen by the ModelFinder implementation available in the program. Inference of the timescales and evolutionary rates were carried out using the TreeDater R package (Volz and Frost, 2017), using the *dater* function. The position of the root node was also inferred in TreeDater. We measured both the age of the stem and crown nodes for areas *C* and *D* (Figure 1).

Analysis of the performance of evolutionary inference on the four scenarios was implemented by comparing features that are relevant for health policy evaluation and decision making: (1) the ages of the epidemic (the root node) and of the transmission events, and (2) the frequency in which the genetic diversity in areas *C* and *D* were recovered as monophyletic. Features (1) were retrieved from the TreeDater output. The frequency of the monophyly in areas *C* and *D* was measured using the built-in functions available in the ape R package.

To evaluate how the timescale of the epidemic affected the general shape of the tree topologies, we calculated measures of tree shape available in the RPANDA R package (Morlon *et al*., 2016), which estimates the spectral density of phylogenies from the normalized modified graph Laplacean (Lewitus and Morlon, 2016). The following shape metrics were computed from the spectral densities: the asymmetry, peakedness, principal eigenvalue and the modality (eigengap) - see Lewitus and Morlon (2016) for details. Distances between spectral densities of topologies were calculated using the Jensen-Shannon (J-S) distance metric using the *JSDtree* function in RPANDA. We used the spectral density metrics to arrange all the topologies simulated into *k* groups (clusters) using the *k*-means clustering analysis. The number of clusters was chosen using the gap statistic (Tibshirani *et al*., 2001). A total of four clusters were found to optimize within- to between-groups J-S distances. We thus assigned topologies to one of these k=4 classes.

### Comparative analysis with SARS-CoV-2 and other epidemics

In order to investigate the effects of the timescale on evolutionary parameters inferred with empirical data, we compared our simulations with the 2019-2020 outbreak of SARS-CoV-2, which consists of the main pandemic of the 21^st^ century so far. By using metrics of topological shape, we evaluated SARS-CoV-2 phylogenetic trees in light of the scenarios simulated. We also compared SARS-CoV-2 topologies with other recent viral outbreaks (SARS in 2003-2004; influenza H1N1 in 2009, and the 2014 Ebola virus outbreak), as well as long term circulating virus species (HIV-1B, DENV-1, and the HCV-1a).

### Sequences and alignments

We downloaded 358 SARS-CoV-2 genomes available in GenBank (Table S1). These genomes cover a broad geographical distribution and were sampled from December 2019 to March 2020. The open reading frames were extracted from the genomes and were subsequently aligned individually. The following genomic regions were analyzed - structural proteins S, E, M, and N, and ORFs 1ab, 3a, 6, 7a and 8. Alignments were carried out on with the MUSCLE software (Edgar, 2004). For other virus lineages, we sampled timescales that covered both short- and long-term infections of human populations. This interval ranged from several months within a single year (SARS-CoV, H1N1, and EBOV) to several decades (HIV-1B). Genome sampling was conducted so as to obtain sequences with collection dates that were evenly spaced in time. Empirical datasets, with the exception of SARS-CoV-2, were downloaded from the Virus Pathogen Resource database (viprbrc.org). Accession numbers were provided in Table S2.

### Phylogenetic inference and molecular dating

All tree topologies were inferred under the maximum likelihood (ML) framework implemented in the IQ-TREE software (Nguyen *et al*., 2014). Model choice was performed automatically in IQ-TREE using the ModelFinder method (Kalyaanamoorthy *et al*., 2017). Following ML tree reconstruction, node ages were estimated with the TreeDater R package (Volz and Frost, 2017) using the *dater* function, and allowing the root node to be inferred.

## Results

### Simulation

**Figure 2 -:**
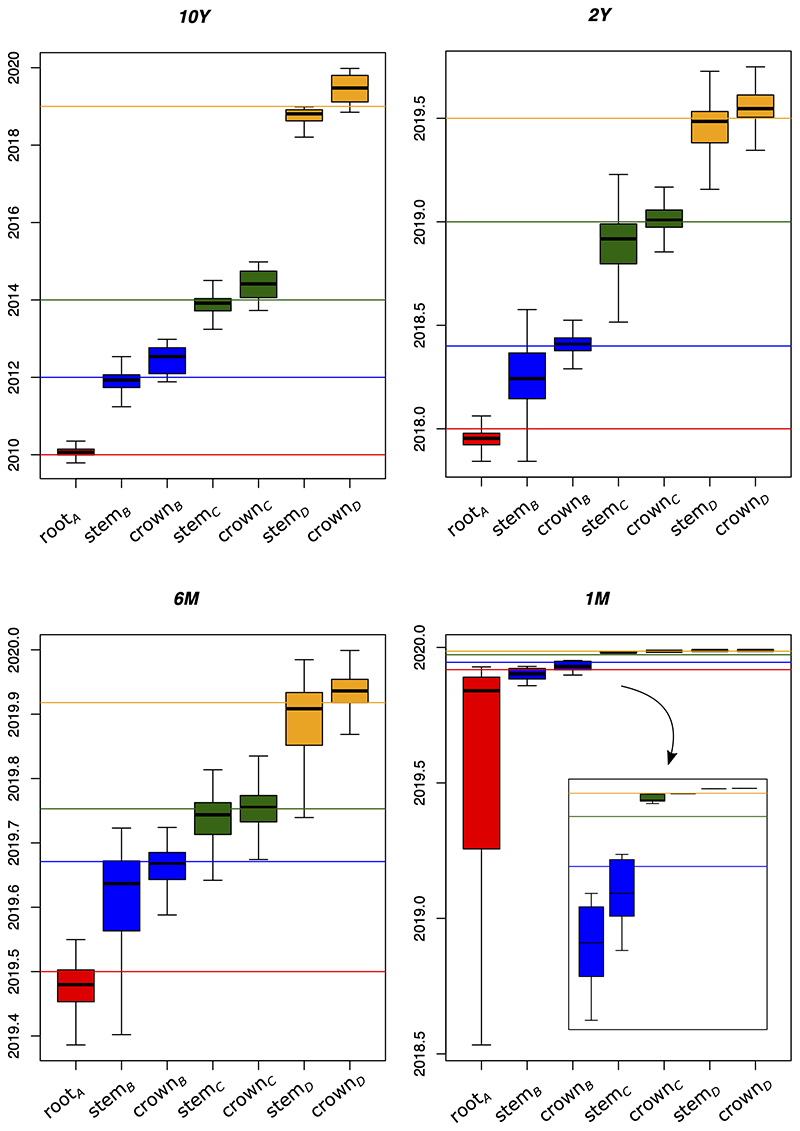
Distribution of the inferred ages of stem and crown nodes of each population (areas *B*-*D*), as well as the age of population in area *A* (root), for each simulated scenario. Horizontal lines are the true simulated ages; for *B*-*D* they represent the true transmission times.

Our simulation showed that the timescale of the epidemic significantly impacted the estimates of evolutionary parameters of epidemiological interest (Figure 2). The age of the transmission events between populations as well as the age of the most recent common ancestor of the pandemic (the age of the root) was most accurately estimated in the *10Y* scenario, in which the errors associated with the estimates were <5% of the total duration of the epidemic (10 years) (Table 1). It is clear that the ages of the stem nodes were better approximations of the true ages of the transmission events than the ages of the crown nodes. Using the ages of the stem nodes, errors associated with the estimates ranged from 0.9% to 3.1% of the total time duration, whereas the crown node yielded 4.1% to 4.8%. Under this scenario, the age of the root was also estimated accurately (0.7% error). As the total duration of the epidemic narrowed, the age of the root became increasingly harder to estimate; the mean difference between the estimates and the true ages shifted from 0.9% (*10Y*) to 1157.2% (*1M*) of the root age (Table 1). In shorter timescales (*6M* and *1M*), the best approximations of the ages of transmission events were inconsistent, because in four cases the ages of the crown nodes were closer to the true value, whereas the remaining two cases were best inferred by the ages of the stem nodes. In most cases, the true transmission ages lied between the estimated ages of the stem and crown nodes. The exception was the *1M* scenario, in which the estimated stem and crown nodes did not bound the true value (Figure 2).

**Table 1 -:** Difference between the average age estimated and the true age for each population (*A* to *D*). Differences were normalized (in percent) by the true age of the root in each scenario.

Scenario	**area *A* (root)**	**area *B* (stem)**	**area *B* (crown)**	**area *C* (stem)**	**area *C* (crown)**	**area *D* (stem)**	**area *D* (crown)**
*10Y*	0.7%	0.9%	4.8%	1.2%	4.1%	3.1%	4.6%
*2Y*	2.5%	1.8%	10.5%	6.3%	1.0%	3.2%	3.1%
*6M*	5.0%	11.6%	1.8%	3.3%	0.2%	7.9%	3.8%
*1M*	1157.2%	65.6%	23.8%	12.0%	14.4%	0.4%	2.4%

The frequency in which the reconstructed genealogy of alleles embedded in populations was monophyletic was also impacted by the timescale of the pandemic (Table 2). In both populations from areas *C* and *D*, shorter timescales resulted in lower frequency of monophyly, indicating that inferred ML gene genealogies would suggest incorrectly that these populations have multiple origins. While in the *10Y* scenario, allelic diversity from areas *C* and *D* were recovered as monophyletic in >90% of the replicates, in the *1M* scenario this figure dropped to 78.3% (area 3) and 89.7% (area *D*).

**Table 2 -:** Frequency in which the reconstructed genealogy of alleles from populations *C* and *D* was monophyletic.

Scenario	**area *C***	**area *D***
*10Y*	94.2%	97.6%
*2Y*	97.3%	93.9%
*6M*	81.9%	93.7%
*1M*	78.3%	89.7%

**Figure 3 -:**
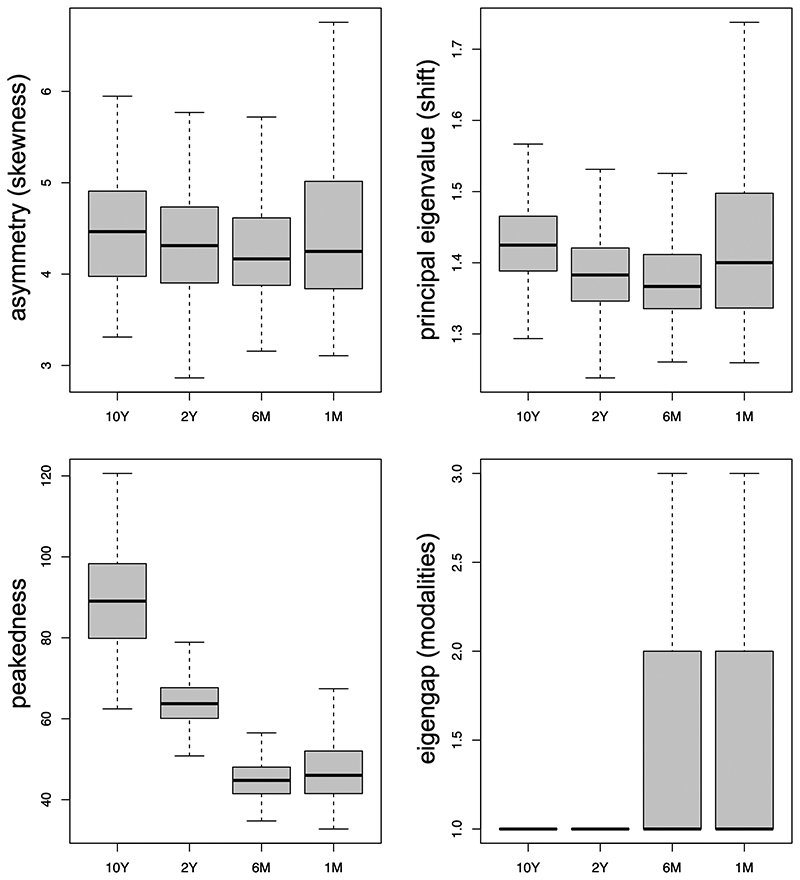
Distribution of metrics of the spectral densities of tree topologies for each scenario.

The metrics calculated from the spectral densities of tree topologies were also affected by the timescales of the epidemics. The asymmetries (skewness) of the density profiles were similar among the trees from the four scenarios investigated (Figure 3), whereas the shift of the profile (the principal eigenvalue) tended to decrease with shorter timescales. In the *1M* scenario, this metric varied significantly between topologies (Figure 3). The two most informative topological metrics to differentiate the scenarios were the peakedness and the number of peaks (the eigen gap, or the number of modalities) of the density profiles (Figure 3). Similar to the shift metric, the peakedness of topologies tended to decrease in shorter timescales, and the variance of the metric was larger in both *10Y* and *1M* scenarios. The number of peaks (modes of evolution) was 1 in most topologies from the *10Y* and *2Y* datasets. In both *6M* and *1M* scenarios, the average number of peaks was 1.4, indicating incorrectly the existence of multiple modes of diversification in a single tree topology.

**Figure 4 -:**
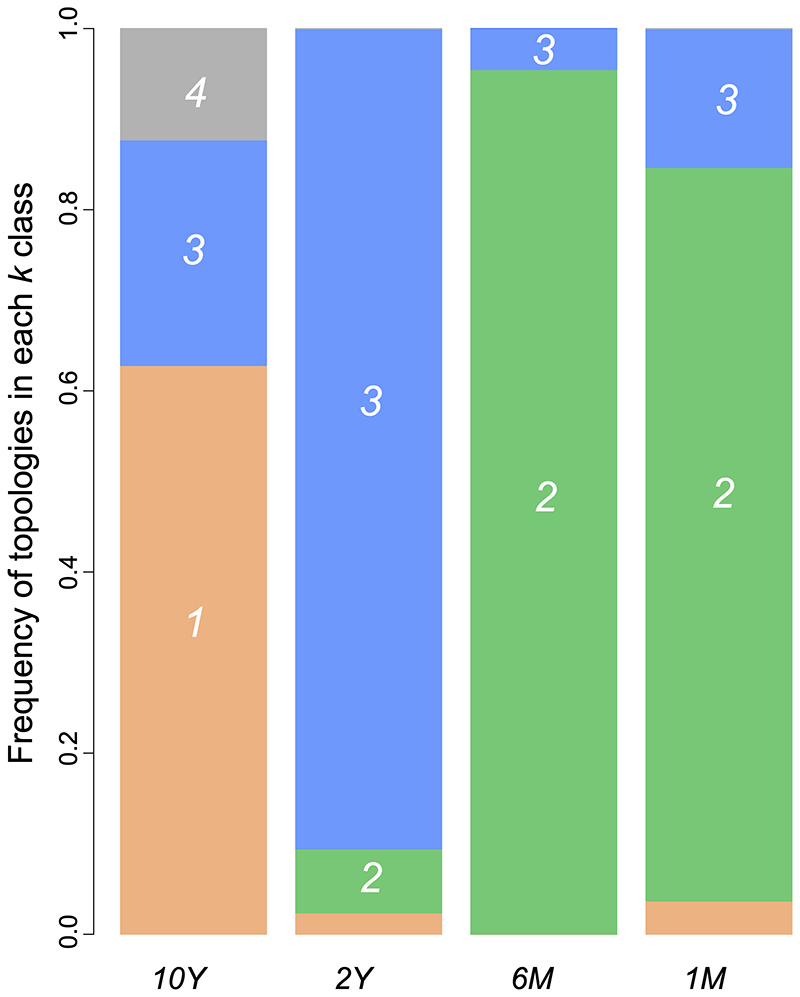
Profiles of cluster assignments of topologies to each of the *k*=4 class in each scenario. Tree topologies from *10Y*, *2Y*, *6M*, and *1M* scenarios were assigned to four clusters (exhibited by colors salmon, green, blue, and gray) using the *k*-means algorithm. In each scenario, bars represent the frequency of topologies in each class.

The gap statistic employed to identify the number of clusters to be used by the *k*-means algorithm was 4. When features measured from the spectral densities were used to cluster the topologies from the several scenarios an interesting picture emerged. The *10Y* and the *2Y* scenarios were distinguished from the shorter timescale scenarios (Figure 4). In *6M* and *1M*, the majority of topologies were assigned to the same cluster (cluster 2, green, Figure 4). A small amount of the *2Y* topologies were also assigned to this cluster, whereas near zero topologies from the *10Y* scenario were assigned to cluster 2. On the other hand, most *10Y* trees were assigned to cluster 1 (salmon, Figure 4), which was almost exclusively found in *10Y* trees. Overall, *10Y* and *2Y* topologies generated distinct cluster assignment profiles that were also distinct from the *6M* and *1M* profiles. The Jensen-Shannon distances between spectral densities of topologies corroborated the cluster profiles (Table 3). As timescales narrowed, J-S distances tended to increase when compared to the *10Y* trees. Therefore, the mean distance between trees from the *10Y* and *1M* scenarios was the highest inter-scenarios distance. It is worth noting that the *10Y* scenario was the case with the highest intra-scenario mean distance.

**Table 3 -:** Mean Jensen-Shannon distances between spectral densities of tree topologies from the modified Laplacean graph. Main diagonal values in bold indicate the mean J-S distance within each population.

	*10Y*	*2Y*	*6M*	*1M*
*10Y*	**1.31**	0.87	0.89	1.19
*2Y*	0.87	**0.33**	0.34	0.78
*6M*	0.89	0.34	**0.33**	0.77
*1M*	1.19	0.78	0.77	**0.56**

### Empirical comparative analysis

**Figure 5 -:**
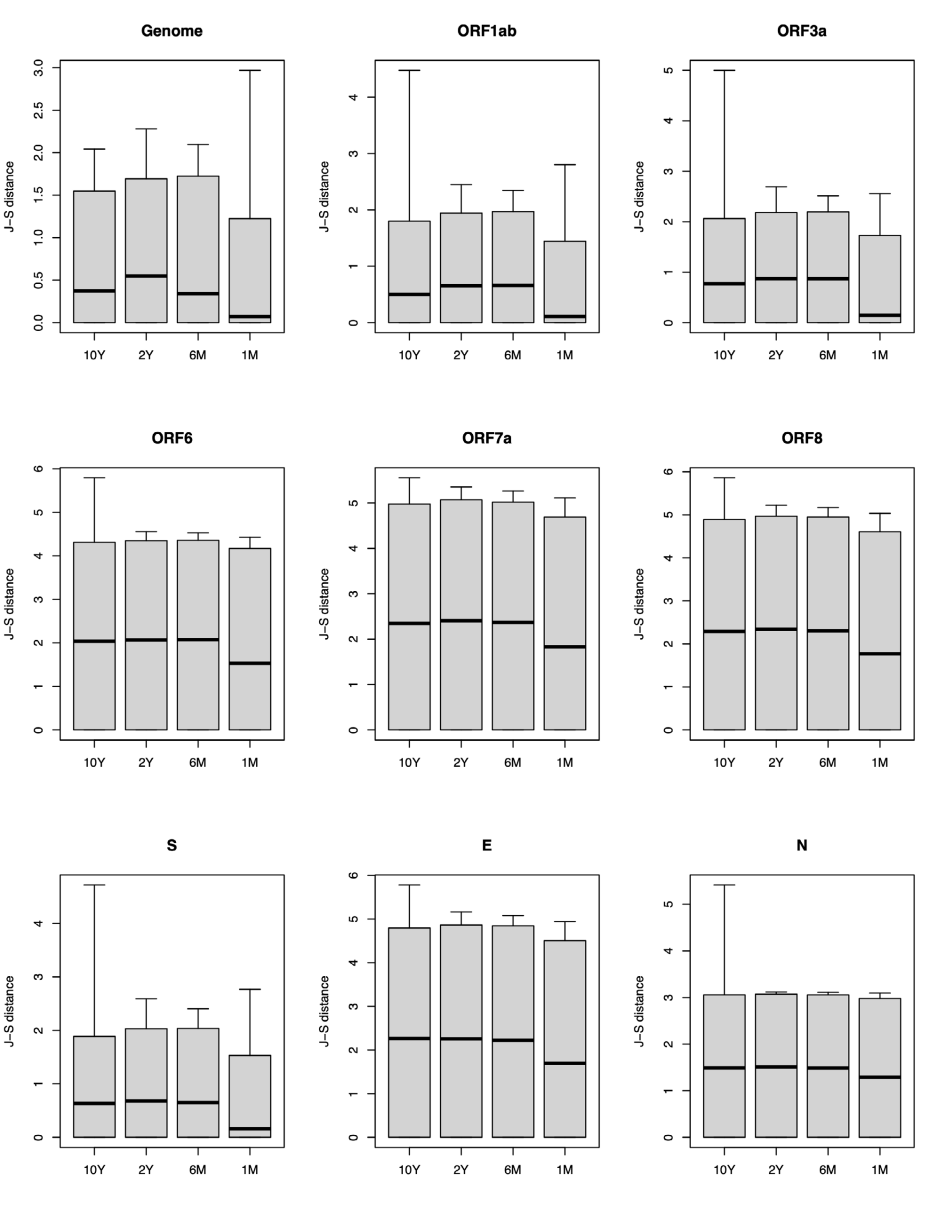
Comparison between the spectral density of SARS-CoV-2 phylogeny for each genomic ORF with the spectral densities of topologies simulated in each scenario. Comparisons were carried out using the Jensen-Shannon (J-S) distance.

We estimated that SARS-CoV-2 genetic diversity coalesced in 20 September 2019, with 95% confidence interval ranging from 14 November 2018 to 16 January 2020. The genome-wide substitution rate was 1.24 x 10^-4^ subst./site/year (5.72 x 10^-5^ - 2.57 x 10^-4^). The inferred position of the root node separated a Chinese sample from the remaining SARS-CoV-2 genomes. The comparison between the spectral densities of simulated trees with SARS-CoV-2 tree topologies estimated for both the entire genome and each ORF independently indicated that the topologies from the *1M* scenario exhibited lower J-S distance for all SARS-CoV-2 genomic regions (Figure 5). SARS-CoV-2 trees were then closer to short-term epidemic scenarios.

**Figure 6 -:**
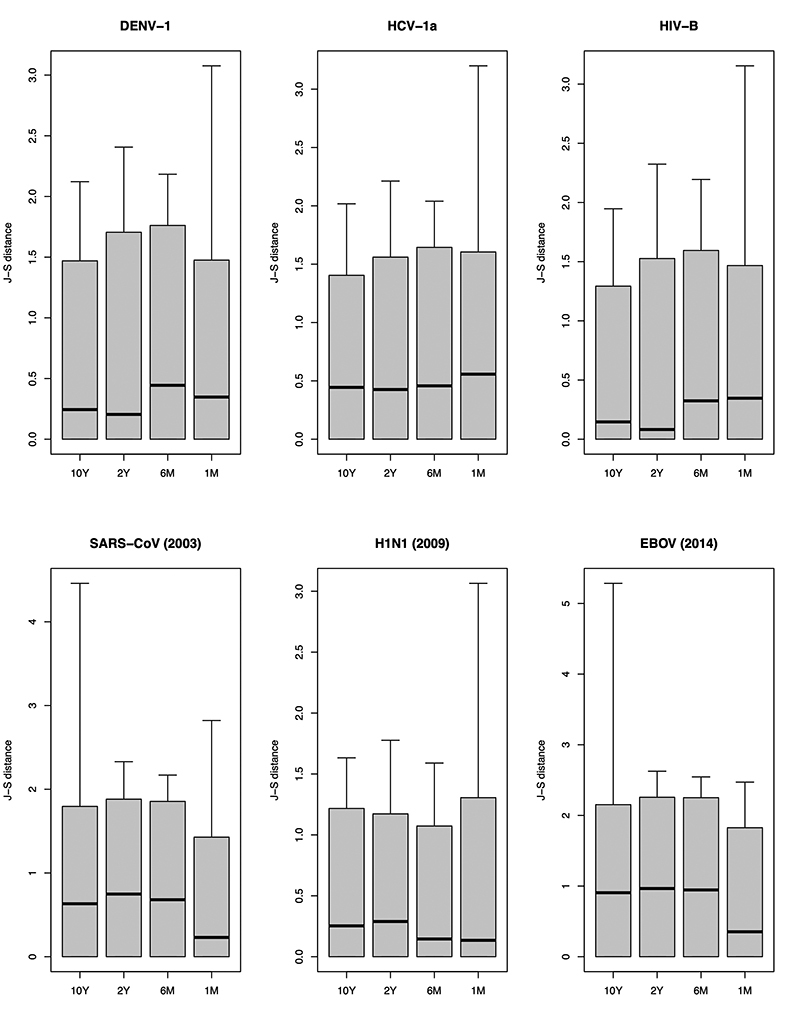
Comparisons between the spectral densities of phylogenies from several virus epidemics with the spectral densities of topologies simulated in each scenario. Comparisons were carried out using the Jensen-Shannon (J-S) distance. DENV01, HCV-1a, and HIV-1B are examples of long-term circulating infections, whereas SARS-CoV (2003), H1N1 (2009), and EBOV (2014) illustrate short-term infections.

When our simulations were evaluated against other virus epidemics, it was possible to differentiate long- and short-term circulating infections (Figure 6). The spectral densities of long-term circulating virus topologies (DENV-1, HCV-1a and HIV-1B) exhibited higher similarity to the *10Y* and *2Y* scenarios, whereas short-term circulating infections were closer to the *6M* and *1M* topologies (SARS-CoV, H1N1, and EBOV). Therefore, the shape of tree topologies contained information on the age of the epidemic.

## Discussion

Our simulations brought forth a number of shortcomings that arise when analyzing the dynamics of virus epidemics within a short timescale. If the age of the circulating infection is young, the inference of evolutionary parameters will suffer from reduced accuracy and precision. For all parameters investigated (the age of the transmission events between geographic areas, the age of the root, and the frequency of monophyly), the scenarios of shorter timescales yielded estimates that deviated more from the true values.

When inferring the ages of transmission events, a simple strategy that improves the success rate of recovering the true age is to employ a bracketing approach, in which both the ages of the crown and stem nodes are communicated. However, when the genetic diversity of the epidemic finds its common ancestor within few generations ago, the interval between the ages of the crown and stem nodes might fail to encompass the true ages. In fact, in such scenarios, the age of the common ancestor of the epidemic (root node) is also harder to estimate, as well as the dynamics of viruses in space. In long-term epidemic scenarios, however, the age of the stem node is the best approximation of the true transmission times, although the age of crown node is frequently used as an estimate of this parameter.

We enumerate two major factors that explain the poor performance of molecular dating methods in short-term epidemic scenarios. Firstly, the low genetic diversity of recent epidemics. If mutations accumulate randomly at a fixed rate, which was the assumption employed to simulate our sequences, then at short time intervals the probability of observing new mutations is lower. The intervals between coalescence events, which define the internal branch lengths, will likely have zero length when measured in units of substitutions/site. Therefore, the structure of the virus phylogeny will be largely random, because sequences lack information on the evolutionary history of virus populations. Such problem could be alleviated by sampling more sites of the virus genome. Assuming that mutation rate is homogeneous across sites, sampling more sites will increase the probability of finding substitutions shared by more than two sequences (internal branches). However, because virus genomes are limited in length, this may not be feasible.

Another factor affecting the inference of evolutionary parameters is the variance of the coalescent process. Because the tree topology of the genealogy of genomes may be different from the phylogeny of virus populations containing the transmission dynamics - the well documented species tree/gene trees discordance (Maddison, 1997; Edwards, 2009) (Figure 1) - parameters associated with transmission times and spatial dynamics may be biased even when sampling an infinite number of nucleotide sites. The finding that monophyly of the genetic diversity within populations may not be recovered even with a single founder virus genome exemplifies how hard it is to infer the spatial dynamics of a recent pandemic. The difficulty in estimating evolutionary parameters will obviously increase in more complex epidemiological scenarios, such as recurrent gene flow between areas (multiple transmission events) and differential selection along the genome (Leaché *et al*., 2014; Solís-Lemus *et al*., 2016). Ideally, the variance of the serially-sampled coalescent should be incorporated when estimating epidemiologic parameters, in way similar to the modeling of gene trees in species tree accomplished by multispecies coalescent (Drummond *et al*., 2003; Biek *et al*., 2015). 

Our results demonstrated that the shape of serially-sampled virus phylogenies, as approximated by the spectral density of the tree, provides information on the temporal dimension - long- and short-term epidemics yielded distinct spectral density profiles. This observation, attained with simulated datasets, was replicated with empirical data. Samples from long-term circulating viruses were similar to long-term simulated datasets as well. We showed that SARS-CoV-2 was most closely related to *1M* trees. This suggests that the SARS-CoV-2 dataset may be as challenging as the *1M* simulation scenario to estimate evolutionary parameters. 

Our estimated age of the common ancestor of the new coronavirus pandemic, 20 Sept. 2019, lies within the uncertainty region defined by the confidence and credibility intervals of previous studies. For instance, although Lai *et al*. (2020) estimated the SARS-CoV-2 TMRCA at 18 Nov. 2019, and Li *et al*. (2020) at 11 Dec. 2019, if confidence intervals are accounted for, one cannot rule out the hypothesis that these estimates are statistically equivalent. The same applies to the evolutionary rate of SARS-CoV-2. As shown in our simulations, this higher uncertainty is expected in short-term epidemic scenarios. Our estimate, 1.24 x 10^-4^ subst./site/year, is in agreement with the values calculated for RNA virus along the last decades (Peck and Lauring, 2018), suggesting that SARS-CoV-2 evolutionary rate is not unique. Unfortunately, the bracketing approach for establishing transmission times is not applicable to the origin of the SARS-CoV-2 pandemic, because its closest known sister lineage, the RaTG13 sequence is evolutionarily distant, artificially increasing the lower bound for the age of the common ancestor (stem node) (Boni *et al*., 2020). However, the strategy can be used to evaluate the age of transmission events along SARS-CoV-2 diversification. Recently, Candido *et al*. (2020) inferred the age of the common ancestor of the earliest among the largest of the Brazilian clade at 22 Feb 2020 (crown node). Following the rationale developed here, we argue that the uncertainty of this estimate should also include the age of the stem node, which would push the entry time of the ancestor of this clade into Brazil to early February. 

The fact that tree shape can distinguish between temporal duration of epidemics is useful as an auxiliary analytical tool. Although the effect of the temporal scale in tree topologies is ultimately dictated by the evolutionary rate, the impact on the estimation of evolutionary parameters may be evaluated directly with ML trees by using metrics of tree shape, no molecular dating is required. The correlation between tree shape and the efficiency in inferring timescales and geographical structure is also useful for conducting sophisticated statistical learning analyses (Sheehan and Song, 2016). For instance, by measuring simple tree shape metrics from the spectral density, we can calculate the probability that the timescale will be recovered correctly. 

In conclusion, we demonstrated that the age of epidemic significantly impacted the inference of evolutionary parameters that are relevant to decision making by health agencies, such as the timescale of virus evolution and the spatial dynamics of the virus populations. In short-term virus epidemics, the probability of recovering the true history of virus populations, as displayed in fully dichotomic tree topologies, is reduced. Metrics of tree topology shape are thus a useful proxy for evaluating the probability that evolutionary parameters were accurately inferred. We showed that short-term epidemics (SARS-CoV-2, SARS 2003, H1N1 2009, EVOV 2014) could be distinguished from long-term circulating viruses (HIV-1B, HCV-1a and DENV-1). Metrics of SARS-CoV-2 phylogeny were similar to simulated scenarios of very recent population dynamics, which resulted in parametric estimates with large uncertainty. Health policies drawn from SARS-CoV-2 evolutionary estimates should thus be designed cautiously. In this sense, simple strategies may be useful, as accounting for both the ages of stem and crown nodes to approximate transmission times.

## Supplementary material

The following online material is available for this article:

Table S1 - Accession numbers, collection dates, and geographical information of SARS-CoV-2 genomes used in this study.

Table S2 - List of Virus Pathogen Resource (www.viprbrc.org) accession numbers of the several human virus genomes used in this study.
