# Supplementary material for: Challenges in estimating virus divergence times in short epidemic timescales with special reference to the evolution of SARS-CoV-2 pandemic
Source: Genet Mol Biol. 2021 Feb 8;44(1 Suppl 1):e20200254. doi: 10.1590/1678-4685-GMB-2020-0254 (PMC7869796; doi:10.1590/1678-4685-GMB-2020-0254)
Supplement: Table S1 - [file 1415-4757-GMB-44-1-s1-e20200254-s1.pdf]

*Genetics and Molecular Biology*

https://doi.org/10.1590/1678-4685-GMB-2020-0254

# Supplementary Material to "Challenges in estimating virus divergence times in short epidemic timescales with special reference to the evolution of SARS-CoV-2 pandemic"

**Table S1** - Accession numbers, collection dates, and geographical information of SARS-CoV-2 genomes used in this study. Calendar years were transformed into decimal system by using 365 days as one unit.

| Accession number | Collection date | Geography |
|---|---|---|
| LC528232 | 2020.112 | China |
| LC528233 | 2020. 112 | China |
| LC529905 | 2020.04109589041 | Japan |
| LC534419 | 2020.18630136986 | Japan |
| MN975262 | 2020.0301369863 | China |
| MT007544 | 2020.06849315068 | Australia |
| MT019529 | 2019.97808219178 | China |
| MT019530 | 2019.99726027397 | China |
| MT019531 | 2019.99726027397 | China |
| MT019532 | 2019.99726027397 | China |
| MT019533 | 2020.00273972603 | China |
| MT039873 | 2020.05479452055 | China |
| MT039890 | 2020.04109589041 | South |
| MT049951 | 2020.04657534247 | China |
| MT066156 | 2020.08219178082 | Italy |
| MT066175 | 2020.08493150685 | Taiwan |
| MT066176 | 2020.09863013699 | Taiwan |
| MT072688 | 2020.03561643836 | Nepal |
| MT123290 | 2020.09863013699 | China |
| MT123291 | 2020.07945205479 | China |
| MT123292 | 2020.07397260274 | China |
| MT123293 | 2020.07945205479 | China |
| MT152824 | 2020.15068493151 | USA |
| MT192759 | 2020.06849315068 | Taiwan |
| MT192765 | 2020.19178082192 | USA |
| MT192772 | 2020.0602739726 | Viet |
| MT192773 | 2020.0602739726 | Viet |
| MT226610 | 2020.05479452055 | China |
| MT233519 | 2020.15890410959 | Spain |
| MT233520 | 2020.15616438356 | Spain |
| MT233521 | 2020.15890410959 | Spain |

| Accession number | Collection date | Geography |
|---|---|---|
| MT233522 | 2020.16712328767 | Spain |
| MT233523 | 2020.17260273973 | Spain |
| MT240479 | 2020.17260273973 | Pakistan |
| MT246449 | 2020.19726027397 | USA |
| MT246450 | 2020.19726027397 | USA |
| MT246451 | 2020.19726027397 | USA |
| MT246452 | 2020.19726027397 | USA |
| MT246453 | 2020.19726027397 | USA |
| MT246454 | 2020.19452054795 | USA |
| MT246455 | 2020.19452054795 | USA |
| MT246457 | 2020.19452054795 | USA |
| MT246458 | 2020.19452054795 | USA |
| MT246459 | 2020.19452054795 | USA |
| MT246460 | 2020.19452054795 | USA |
| MT246461 | 2020.19726027397 | USA |
| MT246462 | 2020.19726027397 | USA |
| MT246463 | 2020.19726027397 | USA |
| MT246464 | 2020.19726027397 | USA |
| MT246465 | 2020.19726027397 | USA |
| MT246466 | 2020.19726027397 | USA |
| MT246467 | 2020.2 | USA |
| MT246468 | 2020.2 | USA |
| MT246469 | 2020.2 | USA |
| MT246470 | 2020.19726027397 | USA |
| MT246471 | 2020.2 | USA |
| MT246472 | 2020.19452054795 | USA |
| MT246473 | 2020.2 | USA |
| MT246474 | 2020.2 | USA |
| MT246475 | 2020.19726027397 | USA |
| MT246476 | 2020.19726027397 | USA |
| MT246477 | 2020.2 | USA |
| MT246478 | 2020.2 | USA |
| MT246479 | 2020.19726027397 | USA |
| MT246480 | 2020.19726027397 | USA |
| MT246481 | 2020.19726027397 | USA |
| MT246482 | 2020.20273972603 | USA |
| MT246484 | 2020.20273972603 | USA |
| MT246485 | 2020.20273972603 | USA |
| MT246486 | 2020.2 | USA |
| MT246487 | 2020.20273972603 | USA |
| MT246488 | 2020.2 | USA |
| MT246489 | 2020.2 | USA |
| MT246490 | 2020.20273972603 | USA |

| Accession number | Collection date | Geography |
|---|---|---|
| MT251972 | 2020.2 | USA |
| MT251974 | 2020.19452054795 | USA |
| MT251975 | 2020.2 | USA |
| MT251976 | 2020.2 | USA |
| MT251977 | 2020.2 | USA |
| MT251978 | 2020.2 | USA |
| MT251979 | 2020.2 | USA |
| MT251980 | 2020.19726027397 | USA |
| MT253696 | 2020.06301369863 | China |
| MT253697 | 2020.06301369863 | China |
| MT253698 | 2020.06301369863 | China |
| MT253699 | 2020.06575342466 | China |
| MT253700 | 2020.06849315068 | China |
| MT253701 | 2020.05753424658 | China |
| MT253702 | 2020.05753424658 | China |
| MT253703 | 2020.06849315068 | China |
| MT253704 | 2020.06849315068 | China |
| MT253705 | 2020.0602739726 | China |
| MT253706 | 2020.0602739726 | China |
| MT253707 | 2020.06849315068 | China |
| MT253708 | 2020.05753424658 | China |
| MT253709 | 2020.05753424658 | China |
| MT253710 | 2020.05753424658 | China |
| MT256924 | 2020.19178082192 | Colombia |
| MT258377 | 2020.21095890411 | USA |
| MT258378 | 2020.21095890411 | USA |
| MT258379 | 2020.21095890411 | USA |
| MT258380 | 2020.21095890411 | USA |
| MT258381 | 2020.21095890411 | USA |
| MT258382 | 2020.21095890411 | USA |
| MT258383 | 2020.21095890411 | USA |
| MT259235 | 2020.19726027397 | USA |
| MT259236 | 2020.20547945205 | USA |
| MT259237 | 2020.20547945205 | USA |
| MT259239 | 2020.20547945205 | USA |
| MT259240 | 2020.20547945205 | USA |
| MT259241 | 2020.20547945205 | USA |
| MT259243 | 2020.19726027397 | USA |
| MT259244 | 2020.20547945205 | USA |
| MT259245 | 2020.20273972603 | USA |
| MT259246 | 2020.19726027397 | USA |
| MT259247 | 2020.20547945205 | USA |
| MT259248 | 2020.2 | USA |

| Accession number | Collection date | Geography |
|---|---|---|
| MT259249 | 2020.19726027397 | USA |
| MT259250 | 2020.2 | USA |
| MT259251 | 2020.2 | USA |
| MT259252 | 2020.2 | USA |
| MT259253 | 2020.20547945205 | USA |
| MT259254 | 2020.20547945205 | USA |
| MT259256 | 2020.19726027397 | USA |
| MT259257 | 2020.20547945205 | USA |
| MT259258 | 2020.19726027397 | USA |
| MT259260 | 2020.20547945205 | USA |
| MT259261 | 2020.20547945205 | USA |
| MT259262 | 2020.2 | USA |
| MT259263 | 2020.19726027397 | USA |
| MT259264 | 2020.19726027397 | USA |
| MT259265 | 2020.19726027397 | USA |
| MT259266 | 2020.19726027397 | USA |
| MT259267 | 2020.2 | USA |
| MT259268 | 2020.19726027397 | USA |
| MT259269 | 2020.2 | USA |
| MT259271 | 2020.20273972603 | USA |
| MT259273 | 2020.20273972603 | USA |
| MT259274 | 2020.2 | USA |
| MT259275 | 2020.2 | USA |
| MT259276 | 2020.20273972603 | USA |
| MT259277 | 2020.20273972603 | USA |
| MT259278 | 2020.20273972603 | USA |
| MT259279 | 2020.20273972603 | USA |
| MT259280 | 2020.20273972603 | USA |
| MT259281 | 2020.20273972603 | USA |
| MT259282 | 2020.20273972603 | USA |
| MT259284 | 2020.20273972603 | USA |
| MT259285 | 2020.20273972603 | USA |
| MT259286 | 2020.20273972603 | USA |
| MT259287 | 2020.20273972603 | USA |
| MT262896 | 2020.19726027397 | USA |
| MT262897 | 2020.19726027397 | USA |
| MT262898 | 2020.19726027397 | USA |
| MT262899 | 2020.19726027397 | USA |
| MT262900 | 2020.19726027397 | USA |
| MT262901 | 2020.19726027397 | USA |
| MT262902 | 2020.19726027397 | USA |
| MT262903 | 2020.19726027397 | USA |
| MT262904 | 2020.19726027397 | USA |

| Accession number | Collection date | Geography |
|---|---|---|
| MT262905 | 2020.19726027397 | USA |
| MT262906 | 2020.19726027397 | USA |
| MT262907 | 2020.19726027397 | USA |
| MT262908 | 2020.19726027397 | USA |
| MT262909 | 2020.19726027397 | USA |
| MT262910 | 2020.19726027397 | USA |
| MT262911 | 2020.19726027397 | USA |
| MT262912 | 2020.19726027397 | USA |
| MT262913 | 2020.19726027397 | USA |
| MT262914 | 2020.19726027397 | USA |
| MT262915 | 2020.19726027397 | USA |
| MT262916 | 2020.19726027397 | USA |
| MT262993 | 2020.19452054795 | Pakistan |
| MT263074 | 2020.18904109589 | Peru |
| MT263381 | 2020.22465753425 | USA |
| MT263382 | 2020.22465753425 | USA |
| MT263383 | 2020.22739726027 | USA |
| MT263384 | 2020.22465753425 | USA |
| MT263385 | 2020.22465753425 | USA |
| MT263386 | 2020.22191780822 | USA |
| MT263387 | 2020.22465753425 | USA |
| MT263388 | 2020.22465753425 | USA |
| MT263390 | 2020.22739726027 | USA |
| MT263391 | 2020.22739726027 | USA |
| MT263392 | 2020.22739726027 | USA |
| MT263394 | 2020.22191780822 | USA |
| MT263395 | 2020.22739726027 | USA |
| MT263396 | 2020.22191780822 | USA |
| MT263397 | 2020.22739726027 | USA |
| MT263398 | 2020.22739726027 | USA |
| MT263399 | 2020.22739726027 | USA |
| MT263400 | 2020.22465753425 | USA |
| MT263401 | 2020.22465753425 | USA |
| MT263402 | 2020.22465753425 | USA |
| MT263403 | 2020.21643835616 | USA |
| MT263404 | 2020.22739726027 | USA |
| MT263405 | 2020.21917808219 | USA |
| MT263406 | 2020.21917808219 | USA |
| MT263407 | 2020.22191780822 | USA |
| MT263408 | 2020.22739726027 | USA |
| MT263409 | 2020.22191780822 | USA |
| MT263410 | 2020.22739726027 | USA |
| MT263411 | 2020.22739726027 | USA |

| Accession number | Collection date | Geography |
|---|---|---|
| MT263412 | 2020.22191780822 | USA |
| MT263414 | 2020.22739726027 | USA |
| MT263415 | 2020.22739726027 | USA |
| MT263416 | 2020.21917808219 | USA |
| MT263417 | 2020.22739726027 | USA |
| MT263419 | 2020.21643835616 | USA |
| MT263422 | 2020.22739726027 | USA |
| MT263423 | 2020.22465753425 | USA |
| MT263424 | 2020.22739726027 | USA |
| MT263425 | 2020.22739726027 | USA |
| MT263426 | 2020.22739726027 | USA |
| MT263427 | 2020.22739726027 | USA |
| MT263428 | 2020.22739726027 | USA |
| MT263429 | 2020.22739726027 | USA |
| MT263430 | 2020.22739726027 | USA |
| MT263431 | 2020.22739726027 | USA |
| MT263432 | 2020.22739726027 | USA |
| MT263433 | 2020.22739726027 | USA |
| MT263434 | 2020.22739726027 | USA |
| MT263435 | 2020.22739726027 | USA |
| MT263436 | 2020.22739726027 | USA |
| MT263437 | 2020.22739726027 | USA |
| MT263438 | 2020.22739726027 | USA |
| MT263439 | 2020.22739726027 | USA |
| MT263440 | 2020.22739726027 | USA |
| MT263441 | 2020.22739726027 | USA |
| MT263442 | 2020.22739726027 | USA |
| MT263444 | 2020.21643835616 | USA |
| MT263446 | 2020.22739726027 | USA |
| MT263447 | 2020.2 | USA |
| MT263448 | 2020.22739726027 | USA |
| MT263449 | 2020.22739726027 | USA |
| MT263450 | 2020.22739726027 | USA |
| MT263451 | 2020.22739726027 | USA |
| MT263452 | 2020.22739726027 | USA |
| MT263453 | 2020.22739726027 | USA |
| MT263455 | 2020.22739726027 | USA |
| MT263456 | 2020.22739726027 | USA |
| MT263457 | 2020.22739726027 | USA |
| MT263458 | 2020.22465753425 | USA |
| MT263459 | 2020.22739726027 | USA |
| MT263460 | 2020.22739726027 | USA |
| MT263461 | 2020.22191780822 | USA |

| Accession number | Collection date | Geography |
|---|---|---|
| MT263462 | 2020.22465753425 | USA |
| MT263463 | 2020.22465753425 | USA |
| MT263464 | 2020.22465753425 | USA |
| MT263465 | 2020.22465753425 | USA |
| MT263466 | 2020.22465753425 | USA |
| MT263467 | 2020.22465753425 | USA |
| MT263468 | 2020.22465753425 | USA |
| MT263469 | 2020.22465753425 | USA |
| MT276323 | 2020.16164383562 | USA |
| MT276324 | 2020.15616438356 | USA |
| MT276325 | 2020.15890410959 | USA |
| MT276326 | 2020.16438356164 | USA |
| MT276327 | 2020.16438356164 | USA |
| MT276328 | 2020.15890410959 | USA |
| MT276329 | 2020.16164383562 | USA |
| MT276330 | 2020.16164383562 | USA |
| MT276331 | 2020.16438356164 | USA |
| MT276597 | 2020.12602739726 | Israel |
| MT276598 | 2020.20273972603 | Israel |
| MT281577 | 2020.18904109589 | China |
| MT291826 | 2019.99726027397 | China |
| MT291827 | 2019.99726027397 | China |
| MT291828 | 2019.99726027397 | China |
| MT291829 | 2019.99726027397 | China |
| MT291830 | 2019.99726027397 | China |
| MT291831 | 2020.06575342466 | China |
| MT291832 | 2020.06849315068 | China |
| MT291833 | 2020.07671232877 | China |
| MT291834 | 2020.07671232877 | China |
| MT291835 | 2020.07397260274 | China |
| MT291836 | 2020.07945205479 | China |
| MT292569 | 2020.18630136986 | Spain |
| MT292570 | 2020.18904109589 | Spain |
| MT292571 | 2020.18630136986 | Spain |
| MT292572 | 2020.18904109589 | Spain |
| MT292573 | 2020.18630136986 | Spain |
| MT292574 | 2020.16712328767 | Spain |
| MT292575 | 2020.18904109589 | Spain |
| MT292576 | 2020.18904109589 | Spain |
| MT292577 | 2020.18356164384 | Spain |
| MT292578 | 2020.18630136986 | Spain |
| MT292580 | 2020.15890410959 | Spain |
| MT292581 | 2020.15890410959 | Spain |

| Accession number | Collection date | Geography |
|---|---|---|
| MT293157 | 2020.24109589041 | USA |
| MT293158 | 2020.24109589041 | USA |
| MT293159 | 2020.24109589041 | USA |
| MT293160 | 2020.24109589041 | USA |
| MT293161 | 2020.24109589041 | USA |
| MT293162 | 2020.24109589041 | USA |
| MT293163 | 2020.24109589041 | USA |
| MT293164 | 2020.24383561644 | USA |
| MT293166 | 2020.24109589041 | USA |
| MT293167 | 2020.24109589041 | USA |
| MT293168 | 2020.24109589041 | USA |
| MT293169 | 2020.24109589041 | USA |
| MT293170 | 2020.24383561644 | USA |
| MT293171 | 2020.24383561644 | USA |
| MT293172 | 2020.24383561644 | USA |
| MT293173 | 2020.24383561644 | USA |
| MT293174 | 2020.23561643836 | USA |
| MT293175 | 2020.24383561644 | USA |
| MT293176 | 2020.23835616438 | USA |
| MT293177 | 2020.23835616438 | USA |
| MT293178 | 2020.23287671233 | USA |
| MT293179 | 2020.23287671233 | USA |
| MT293180 | 2020.23835616438 | USA |
| MT293181 | 2020.23287671233 | USA |
| MT293182 | 2020.23287671233 | USA |
| MT293183 | 2020.23287671233 | USA |
| MT293184 | 2020.23287671233 | USA |
| MT293185 | 2020.23835616438 | USA |
| MT293186 | 2020.23287671233 | USA |
| MT293187 | 2020.23287671233 | USA |
| MT293188 | 2020.23835616438 | USA |
| MT293189 | 2020.23287671233 | USA |
| MT293190 | 2020.23287671233 | USA |
| MT293191 | 2020.23287671233 | USA |
| MT293192 | 2020.23835616438 | USA |
| MT293194 | 2020.24383561644 | USA |
| MT293195 | 2020.23835616438 | USA |
| MT293196 | 2020.24383561644 | USA |
| MT293197 | 2020.24383561644 | USA |
| MT293198 | 2020.24383561644 | USA |
| MT293199 | 2020.24383561644 | USA |
| MT293200 | 2020.24383561644 | USA |
| MT293201 | 2020.24383561644 | USA |

| Accession number | Collection date | Geography |
|---|---|---|
| MT293202 | 2020.24383561644 | USA |
| MT293203 | 2020.24383561644 | USA |
| MT293204 | 2020.24383561644 | USA |
| MT293205 | 2020.24383561644 | USA |
| MT293206 | 2020.24383561644 | USA |
| MT293207 | 2020.24383561644 | USA |
| MT293208 | 2020.24383561644 | USA |
| MT293209 | 2020.24383561644 | USA |
| MT293210 | 2020.24109589041 | USA |
| MT293211 | 2020.23287671233 | USA |
| MT293212 | 2020.24383561644 | USA |
| MT293213 | 2020.24383561644 | USA |
| MT293214 | 2020.23561643836 | USA |
| MT293215 | 2020.23835616438 | USA |
| MT293216 | 2020.23835616438 | USA |
| MT293218 | 2020.24109589041 | USA |
| MT293219 | 2020.24109589041 | USA |
| MT293220 | 2020.23835616438 | USA |
| MT293221 | 2020.24109589041 | USA |
| MT293222 | 2020.24109589041 | USA |
| MT293223 | 2020.24109589041 | USA |
| MT293224 | 2020.24109589041 | USA |
| MT293225 | 2020.24109589041 | USA |
| MT295464 | 2020.18904109589 | USA |
| MT295465 | 2020.23561643836 | USA |
| NC_045512 | 2019.95616438356 | China |
