# Supplementary material for: Challenges in estimating virus divergence times in short epidemic timescales with special reference to the evolution of SARS-CoV-2 pandemic
Source: Genet Mol Biol. 2021 Feb 8;44(1 Suppl 1):e20200254. doi: 10.1590/1678-4685-GMB-2020-0254 (PMC7869796; doi:10.1590/1678-4685-GMB-2020-0254)
Supplement: Table S2 - [file 1415-4757-GMB-44-1-s1-e20200254-s2.pdf]

Genetics and Molecular Biology
https://doi.org/10.1590/1678-4685-GMB-2020-0254

Supplementary Material to "Challenges in estimating virus divergence times in short epidemic timescales with special reference to the evolution of SARS-CoV-2 pandemic"

Table S2 - List of Virus Pathogen Resource (www.viprbrc.org) accession numbers of the several human virus genomes used in this study.

H1V1-B	HCV-1a	DENV-1	SARS-CoV	H1N1	EBOV
MF373174	KY620880	GQ868601	AY559097	GQ894929	EM095
MN486021	EU255999	FJ410186	AY351680	GQ377035	G3676
EF514710	EU255996	FJ205874	NC_004718	GQ117067	G3686
FJ388940	EU155213	FJ562106	AY274119	GQ221788	KJ660346
MK116088	EU482855	JN819417	AY304486	GQ117079	KJ660348
MN090752	EU255989	FJ410190	AY304488	GQ221805	KJ660347
KT124797	EU256004	FJ478458	AY278489	GQ221809	G3680
JQ403053	EU687195	FJ410189	AY390556	GQ232037	G3687
KT276267	EU155314	FJ205872	AY394996	CY043086	G3683
FJ388955	EU255966	KF973459	AY394997	CY043094	EM096
MF109479	EU255970	JN819425	AY395003	CY073394	G3679
KT259308	KC844049	KC692499	AY394995	CY073402	EM098
KJ019215	EU234063	GQ868561	AY394986	GQ280797	EM104
U12055	EU862838	JX669468	AY394994	FJ966082	EM106
MF373185	EU687193	JQ922547	AY394985	GQ117044	G3707
MF373144	EU482857	AB608789	AY278554	KC781723	G3798
EF514697	EU482846	EU482791	AY394993	FJ966960	G3805
MN486001	EU256050	GQ199836	AY304495	KF527476	EM113
MF373125	EU155313	FJ687428	AY394990	KF527477	EM115
MF373161	EU255953	KC762647	AY394989	KF009554	G3713
MN090900	EU255968	MT447147	AY394992	FJ966974	G3845
KC797176	FJ390394	MG840576	AY394983	KC781785	G3846
MN485985	EU255986	MF033220	AY463059	FJ969540	G3764
AY423381	EU155287	MG840561	AY463060	FJ981613	G3817
FJ853622	EU255988	MG840548	AY595412	CY121680	EM111
AY713411	FJ390395	HM181959	AY278487	KU933485	G3750
EU786678	EU155293	FJ182018	AY278490	CY266191	EM112
MN449474	EU255981	GU131729	AY278488	KC780060	EM124
